# Direct Methylation of Benzene with Methane Catalyzed by Co/MFI Zeolite

**DOI:** 10.1002/cctc.201800724

**Published:** 2018-07-11

**Authors:** Koshiro Nakamura, Akihito Okuda, Kiyotaka Ohta, Hitoshi Matsubara, Kazu Okumura, Kana Yamamoto, Ryosuke Itagaki, Satoshi Suganuma, Etsushi Tsuji, Naonobu Katada

**Affiliations:** ^1^ Center for Research on Green Sustainable Chemistry Tottori University 4-101 Koyama-cho Minami Tottori 680-8552 Japan; ^2^ Applied Chemistry Kogakuin University 2665-1 Nakano-cho, Hachioji, Tokyo 192-0015 Japan

**Keywords:** Methane, Methylation, Cobalt, MFI Zeolite, Benzene

## Abstract

Cobalt‐loaded MFI zeolite showed distinct activity for direct methylation of benzene with methane into toluene. High activity was found at around 0.6 of Co/Al molar ratio. Incorporation of carbon from methane into the methyl group of toluene was confirmed with isotope tracer experiments and mass spectroscopy. Ammonia infrared‐mass spectroscopy temperature‐programmed desorption, transmission electron microscopy, X‐ray absorption near edge spectroscopy and extended X‐ray absorption fine structure indicated that Lewis acidic divalent (+II of oxidation state) Co species mono‐atomically dispersed on the ion exchange site of MFI zeolite was the active species.

## Introduction

Conversion of methane into value‐added organic compounds, i. e. alkenes and mono‐cyclic aromatics, has been strongly required.^1^, ^2^, ^3^, ^4^ Considerable efforts have been made in activation of methane,^4^, ^5^, ^6^, ^7^, ^8^, ^9^, ^10^, ^11^, ^12^, ^13^, ^14^ e. g. partial oxidation into methanol,^9^, ^10^, ^11^, ^12^ and aromatization.^14^, ^15^, ^16^, ^17^ Methylation of benzene ring with methane can be an option of effective use of methane, because it can produce such a valued compound as para‐xylene. For example, combining the methylation of benzene into toluene (Reaction (1), shown in Eq. (1)), and shape selective disproportionation of toluene into para‐xylene^18^,^19^ is expected to be a new process for the production of para‐xylene from toluene and methane.

(1) $$\mathrm{CH}{}_{4}+C{}_{6}H{}_{6}\to H{}_{3}C-C{}_{6}H{}_{5}+H{}_{2}$$

In very early studies, formation of methylated product from a mixture of aromatic compound and methane was reported.^20^, ^21^, ^22^, ^23^ However, in the case of production of toluene from benzene and methane, it has been pointed out that toluene was possibly formed by unexpected reactions^24^ like hydrogenolysis of benzene,^25^ where methane acted just as a hydrogen source. Careful experiments using isotope tracers then evidenced that the methylation of benzene with methane (Eq. (1)) was catalyzed on such zeolite‐supported transition metal species as Cu/*BEA^26^ and Ag/MFI.^27^

On the other hand, it was pointed out that equilibrium of Reaction (1) was a problem as follows. The standard enthalpy and entropy are 41.94 kJ mol^−1^ and −4.11 J K^−1^ mol^−1^ (calculated from thermodynamic properties^28^,^29^), respectively, at 298 K where all the reactants and products are gases, approximately equivalent to the standard Gibbs energy +45.1 kJ mol^−1^ and the equilibrium constant 9×10^−4^ at 773 K; details in Figure S1. It has been pointed out that the conversion of Reaction (1) is limited by the low equilibrium constant,^24^ suppressing the efficiency under past economic conditions. However, the recent demand for utilization of methane as stated above encourages re‐investigation of this reaction.

As stated above, Cu/*BEA^26^ and Ag/MFI^27^ were reported to possess the activity for methylation of benzene with methane. The activity was found also on Pt/MFI^30^ and In/MFI.^31^ In addition, it has been known that a similar reaction, oxidative methylation of benzene (Reaction (2), shown in Eq. (2)), was found to proceed on H‐ and Na‐MFI and its ion exchanged formed with Co, Mn and Cu salts;^32^,^33^ the valence of transition elements has not been mentioned in the papers. Also for the partial oxidation of methane into methanol, zeolite‐supported transition metal species were reported to show the catalytic activity.^34^, ^35^, ^36^, ^37^, ^38^ It has been known that zeolite (mainly MFI)‐supported metal species are active also for combustion of methane,^39^ reduction of NO_*x*_ with methane,^40^ aromatization of methane^14^ and the activation of methane at low temperature.^41^ All these studies suggest that zeolite‐supported transition metal species are promising candidates of catalysts for reactions involving the methane activation step.

(2) $$\mathrm{CH}{}_{4}+C{}_{6}H{}_{6}+1/2O{}_{2}\to H{}_{3}C-C{}_{6}H{}_{5}+H{}_{2}O$$

Here we mention the difference between Reaction (1) and (2). Comprehensive studies by Adebajo et al. clarified that many kinds of zeolite‐supported transition metal species (sometimes typical elements) showed catalytic activity for Reaction (2).^13^ Generally, the rate of Reaction (2) is higher than Reaction (1) at the same temperature in similar partial pressures of benzene and methane. However, Reaction (2) consumes the hydrogen to form water whereas Reaction (1) forms hydrogen, and complete oxidation of organic materials into CO_2_ as a side reaction is probably unavoidable in the presence of oxygen. From these viewpoints, the non‐oxidative methylation of benzene with methane (Reaction (1)) should be studied in more detail. Because the rate of Reaction (1) is substantially low, the investigation to find an efficient catalyst should be important compared to the case of Reaction (2).

As stated above, the preceding literatures reported essential activity of Cu/*BEA,^26^ Ag/MFI,^27^ Pt/MFI^30^ and In/MFI^31^ for the methylation of benzene with methane, indicating that unique activity was created by the combination of transition metal species and zeolite. In the case of Ag/MFI and In/MFI, Ag^+^ and InO^+^ species, respectively, held by the ion exchange site of MFI have been identified as the active species.^27^,^31^ It has also been clarified that unique function of the transition metal species concerning the methane activation was induced by the zeolite ion exchange site in other cases.^39^,^41^ We believe that the analysis of structure in atomic dimension and the quantitative analysis of catalytic sites are important in this field for clarifying the role of ion exchange site and unique function of transition metal species on it. X‐ray absorption near edge spectroscopy (XANES) and extended X‐ray absorption fine structure (EXAFS) were utilized to characterize the oxidation state and structure of active species in atomic scale. Ammonia infrared mass spectrometry‐temperature programed desorption (IRMS‐TPD), which was recently developed by our group,^42^ was also applied for the quantitative analysis of Brønsted and Lewis acid sites, showing the quantities of ion exchange sites uncovered and covered by the transition metal species. The analysis of Lewis acidity is also related directly to the activity, because it has been known that Lewis acid strength affects the activity in reactions of methylation reagents.^43^

Based on these backgrounds, we investigate the catalytic performances of various zeolite‐supported transition metal species for the methylation of benzene with methane into toluene in non‐oxidative conditions. The first purpose is the screening of catalysts with wide variation. Most of the preceding papers^26^,^27^,^30^,^31^ reported the catalytic performances for this reaction in batch or closed circular systems, reflecting very slow reaction rate, but a fixed‐bed continuous flow method is here employed for practical investigation. The reaction formula and side reactions are analyzed by using isotope tracers and mass spectroscopy (MS). As the second purpose of the study, the physicochemical properties of active species are analyzed using such advanced techniques as XANES, EXAFS and IRMS‐TPD. To our knowledge, such a comprehensive study has not been done for the methylation of benzene with methane in non‐oxidative conditions. We here report high activity of Co/MFI and the nature of active species.

## Results and Discussion

### Catalytic Activity

**Figure 1:**
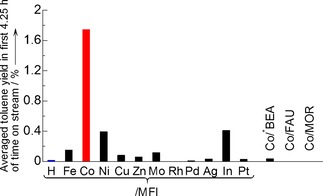
Catalytic activity for methylation of benzene with methane at 773 K, *P*_CH4_=98.6 kPa, *P*_C6H6_=2.7 kPa and *W*_cat_/*F*_benzene_=147 g_cat_ h mol_benzene_^−1^, over zeolite‐supported metal species, taken from data in Figure S2. As Co/MFI, IMP‐Co‐0.6 (see Table S1) was employed; [Al] and Co/Al molar ratio were 1.3 mol kg^−1^ and 0.6, respectively. Compositions and preparation methods of other catalysts are shown in Table S1.

The continuous flow reaction of benzene and methane was examined at 773 K on various zeolite‐supported transition metal species, and the formation of toluene was observed on some catalysts. Figure 1 compares the toluene yield on various elements (including H) impregnated on MFI zeolite, and Co impregnated on various supports. Among the employed catalysts covering various elements and zeolite supports, only Co/MFI showed remarkable activity. Toluene was detected, as well as trace of xylene, while no other organic product was found in the product on Co/MFI. Zeolite (mainly MFI)‐supported Ag, In, Cu, Zn, Pt and Mo have been reported to show activities for the present reaction^26^, ^27^, ^28^, ^29^, ^30^, ^31^ or the reactions/activation of methane, i. e., oxidation of methane to methanol,^34^ combustion of methane,^39^ selective catalytic reduction of NO_*x*_ with methane,^40^ aromatization of methane^14^ and the activation of methane at low temperature.^41^ Co/MFI showed obviously higher activity for methylation of benzene with methane than those on the other catalysts including the above metals loaded on zeolites. Although Baba et al. reported the activity of Ag^+^ species on Ag/MFI for the same reaction (methylation of benzene) at 673 K,^27^ the activity of Ag/MFI was obviously lower than Co/MFI in the present results. On the contrary, Ni/MFI and In/MFI showed small activities, and the latter is in agreement with Gabrienko et al.;^31^ Co/MFI was found to be more active than all of them. On the other hand, Adebajo et al. reported that Co/MFI showed the activity for oxidative methylation of benzene with methane (Eq. (2)), but several other catalysts such as Mn, Cu, and Na‐modified MFI also showed the comparable activity.^32^,^33^ It is noteworthy that the non‐oxidative methylation (Reaction (1)) seems to be substantially difficult and its rate is sensitive to the nature of catalyst compared to Reaction (2), and therefore only Co/MFI had distinguished activity.

To confirm the superiority of Co/MFI for this reaction, the reaction tests were examined also under high pressures of hydrogen and benzene with higher reaction rates. It was demonstrated that Co/MFI always showed high activity compared to the other catalysts (Figure S3).

Preceding literature reported that toluene was formed in the co‐presence of benzene and methane even on H‐MFI without transition metal through the hydrogenolysis of benzene,^25^ but in the present case, the yield of toluene on H‐MFI was negligible as shown in Figure 1. The influence of hydrogenolysis is thus believed to be small in the present conditions, as also evidenced by the experiments using isotope tracers shown later.

**Figure 2:**
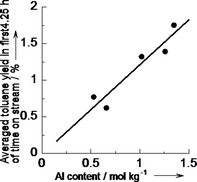
Catalytic activity for methylation of benzene with methane at 773 K, *P*_CH4_=98.6 kPa, *P*_C6H6_=2.7 kPa and *W*_cat_/*F*_benzene_=147 g_cat_ h mol_benzene_^−1^ over Co‐impregnated on MFI with various Al contents with keeping Co/Al molar ratio at 0.6, taken from Figure S2 (a).

Figure 2 shows the influence of Al content of MFI zeolite as the support with keeping the Co/Al molar ratio at 0.6. The yield increased with the Al content up to [Al]=1.3 mol kg^−1^ corresponding to SiO_2_/Al_2_O_3_=22, i. e., the highest Al concentration in MFI commercially available.

**Figure 3:**
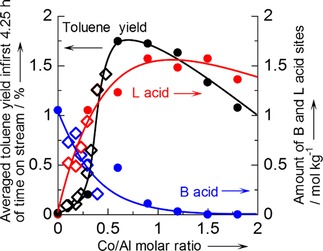
Catalytic activity for methylation of benzene with methane at 773 K, *P*_CH4_=98.6 kPa, *P*_C6H6_=2.7 kPa and *W*_cat_/*F*_benzene_=147 g_cat_ h mol_benzene_^−1^ (• and ◊), taken from Figure S3 (b), and amounts of Brønsted (• and ◊) and Lewis (• and ◊) acid sites on Co/MFI prepared by impregnation (•) and ion exchange (◊) methods using MFI with SiO_2_/Al_2_O_3_=22 ([Al]=1.3 mol kg^−1^) plotted against Co/Al molar ratio.

Figure 3 (• and ◊) shows the influence of Co content on MFI with [Al] fixed at 1.3 mol kg^−1^. Because Co/Al molar ratio obtained by the ion exchange in the present conditions was <0.48, further loading of Co was performed only by the impregnation method, whereas the impregnation (•) and ion exchange (◊) method gave a common relationship between the activity and Co/Al molar ratio <0.48. The activity was negligible at Co/Al=0 under these conditions, and introducing Co created the activity. The toluene yield showed the maximum at Co/Al=0.6, and further loading reduced the activity at Co/Al>0.9.

### Confirmation of Reaction Formula

**Figure 4:**
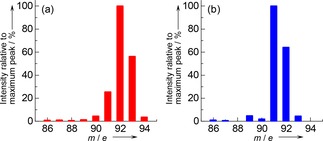
Intensity of peaks in GC‐MS spectra of the outlet liquid after (a) ^13^CH_4_ (^13^C‐enriched methane)+benzene and (b) ^12^CH_4_+benzene reactions on IMP‐Co‐0.6 at *W*_cat_/*F*_benzene_=147 g_cat_ h mol_benzene_^−1^ at 773 K, *P*_CH4_=98.6 kPa, *P*_C6H6_=2.7 kPa and *W*_cat_/*F*_benzene_=147 g_cat_ h mol_benzene_^−1^.

Experiments using isotope tracer were carried out on IMP‐Co‐0.6 to confirm that the toluene formation was ascribed to the Reaction (1) but not due to the benzene hydrogenolysis.^25^ Methane enriched with ^13^C (hereafter ^13^CH_4_) and ordinal benzene (hereafter shown as ^12^C_6_H_6_, but containing naturally abundant ^13^C) were used as the reactants, and the products were analyzed with a gas chromatography‐mass spectrometer (GC‐MS) and ^13^C nuclear magnetic resonance (NMR). Results of the GC‐MS analysis are shown in Figure 4 and indicate that the *m*/*e* ratios of major peaks of toluene produced by the reaction of ^13^CH_4_+^12^C_6_H_6_ were 92 and 93, consistent with the molecular weight 93 of ^13^C^12^C_6_^1^H_8_, whereas the reaction of ^12^CH_4_ (ordinal methane)+^12^C_6_H_6_ gave the *m*/*e* ratios of toluene 91 and 92, showing the molecular weight 92 of ^12^C_7_^1^H_8_.

**Figure 5:**
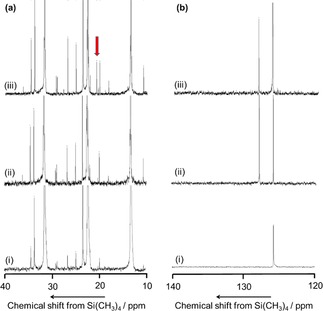
(a) 10–40 ppm and (b) 120–140 ppm regions of ^13^C NMR spectra of (i) blank solution of hexane (solvent) and 1,4‐diisopropylbenzene (inner standard material), (ii) outlet materials of reaction of [^12^CH_4_+^12^C_6_H_6_ (benzene)], and (iii) outlet materials of reaction of [^13^CH_4_+^12^C_6_H_6_] on IMP‐Co‐0.6 at 773 K, *W*_cat_/*F*_benzene_=147 g_cat_ h mol_benzene_^−1^ in 98.6 and 2.7 kPa of methane and benzene, respectively.

Figure 5 shows ^13^C NMR spectra. The naturally abundant ^13^C was found in a mixture of benzene, the solvent (hexane) and the inner standard material (1,4‐diisopropylbenzene) [(i) and (ii)]. In addition to these peaks, a signal at 21.4 ppm assigned to the methyl group of toluene^44^ was observed in the product of ^13^CH_4_+^12^C_6_H_6_ (iii) but not in the product of ^12^CH_4_+^12^C_6_H_6_ (ii) [Figure 5 (a)]. On the other hand, the product of ^13^CH_4_+^12^C_6_H_6_ (iii) showed no peaks at 125.3, 128.3, 129.1 nor 137.8 ppm where carbons in the benzene ring of toluene might show resonances^44^ [Figure 5 (b)]. It has thus been evidenced that most of toluene was formed from a pair of methane and benzene molecules, and the carbon atom in the methyl group of toluene came from methane, whereas the carbon atoms in the benzene ring came from benzene.

**Figure 6:**
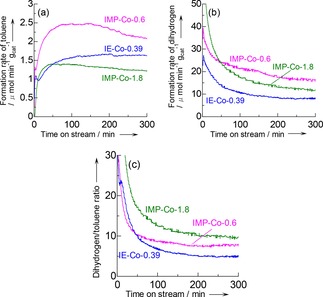
Changes in formation rate of (a) toluene and (b) dihydrogen, and (c) dihydrogen/toluene molar ratio with time on stream in the methylation of benzene with methane at 773 K, *P*_CH4_, *P*_He_ and *P*_C6H6_=91.4, 6.8 and 3.2 kPa, respectively, and *W*_cat_/*F*_benzene_=125 g_cat_ h mol_benzene_^−1^ on Co/MFI. The pretreatment of catalysts for these experiments were carried out at 823 K in nitrogen flow for 2 h.

The MS analysis of the outlet gas detected only dihydrogen (H_2_), toluene and trace of xylene as the products of the reaction of ^12^CH_4_+^12^C_6_H_6_ on Co/MFI, whereas no other hydrocarbon was found at *m*/*e* <100. This is consistent with that the hydrogenolysis of benzene did not occur. Figure 6 shows the rates of formation of toluene and dihydrogen and their time course on IE‐Co‐0.39, IMP‐Co‐0.6 and IMP‐Co‐1.8 as the catalysts; the toluene formation rate was consistent with the experiments shown in Figure 3. The formation of dihydrogen was significant at the initial stage of flow reaction (<50 min) [Figure 6 (b)]. This suggests that the dehydrogenation of methane (3) proceeded on these catalysts. The formation rate of dihydrogen decreased quickly, especially for IMP‐Co‐1.8. Probably the carbonaceous formed due to the reaction shown in Equation (3) blocked the active sites for the dehydrogenation.

After this initial deactivation, the formation of dihydrogen has continued. The molar ratio of dihydrogen/toluene was generally higher than unity even after the initial deactivation, as shown in Figure 6 (c). This ratio should be unity if only Reaction (1) proceeds. The value higher than unity indicates the dehydrogenation shown in Equation (3) is taking place as a side reaction. The ratio was in the order of IMP‐Co‐1.8>IMP‐Co‐0.6>IE‐Co‐0.39, indicating that excess of Co forming the aggregates resulted in the significant side reaction.

(3) $$\mathrm{CH}{}_{4}\to C+2H{}_{2}$$

These findings indicate that the direct methylation of benzene with methane into toluene and dihydrogen (Eq. (1)) proceeded on Co/MFI in the employed reaction conditions, accompanied with dehydrogenation of methane into carbonaceous material and dihydrogen as a side reaction; its extent was dependent on the loading of Co. Simultaneously undesired reactions such as hydrogenolysis of benzene was not observed.

### Investigation of Active Species

The acidic property was analyzed by means of ammonia IRMS‐TPD method.^42^ Figure 3 also shows the amount of the Brønsted and Lewis acid sites as overlapped on the catalytic activity. Similarly, to the activity, the impregnation and ion exchange methods gave no remarkable difference in the relationship between the acid amounts and Co/Al ratio. On the parent H‐MFI (Co/Al=0), a considerable amount of Brønsted acid sites were detected, and the loading of Co decreased the Brønsted acid sites. This indicates that the Co species were bound to the ion exchange sites. The Lewis acid sites were generated by the Co loading, showed the maximum at Co/Al=ca. 0.9, and then gradually decreased. Generation of Lewis acid sites by introduction of transition metal species on the ion exchange site of zeolite has been found.^45^,^46^ As stated in the previous paragraph, the catalytic activity for methylation of benzene with methane was also created by the Co loading, showed the maximum and decreased with further loading. The similar trend suggests that the active site was Lewis acidic Co species held by the ion exchange site.

**Figure 7:**
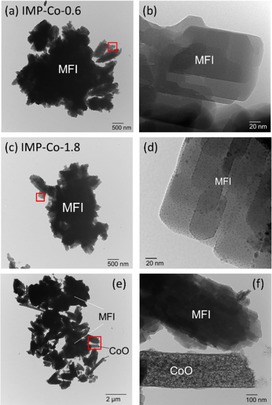
TEM images of (a), (b) IMP‐Co‐0.6 and (c)–(f) IMP‐Co‐1.8.

**Figure 8:**
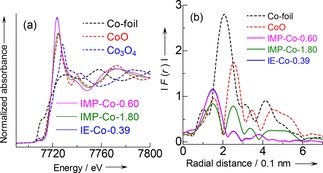
(a) XANES spectra of Co/MFI pretreated under reaction conditions (in nitrogen flow at 823 K for 1 h) and reference samples, and (b) radial distribution function of Co K‐edge EXAFS of Co/MFI under reaction conditions (in nitrogen flow at 823 K for 1 h) and reference samples.

Figure 7 shows transmission electron microscope (TEM) images of IMP‐Co‐0.6 and IMP‐Co‐1.8. Both had coffin‐shaped particles, i. e., typical crystallites of MFI type zeolite. Aggregates were scarcely observed on IMP‐Co‐0.6 [Figure 7 (b)], implying that Co species was highly dispersed in the micropores of MFI, because the solution of TEM was ca. 1 nm. On the contrary, IMP‐Co‐1.8 had aggregates on it [Figure 7 (d)], as well as rod‐shaped structures [Figure 7 (f)]. They were identified to fcc‐CoO from the electron diffraction pattern (Figure S5). Figure 8 (a) shows the XANES (X‐ray absorption near edge structure) spectra after the pretreatment in the same conditions to those of reaction (in N_2_ flow at 823 K for 1 h). The spectra of all the Co/MFI samples employed here had similar positions and shapes to those of bulk CoO, indicating that the oxidation state of Co supported on MFI was insensitive to the loading amount and around +II in the experimental region of Co loading. Figure 8 (b) shows the radial distribution function of Co K‐edge EXAFS (extended X‐ray absorption fine structure). The Co/MFI samples with small Co loadings (IMP‐Co‐0.6 obtained by impregnation and IE‐Co‐0.39 obtained by ion exchange) showed a distribution peak at 0.15 nm, attributed to Co−O, whereas the sample with excess of Co (IMP‐Co‐1.8) had an additional peak at 0.25 nm, attributed to Co−O−Co, similarly to the bulk oxides. These indicate that Co species were mono‐atomically dispersed at Co/Al <0.6, whereas a fraction of Co species were oligomerized or aggregated with further loading.

We have to mention that, the efficiency of Co (+II) was thus demonstrated, but we have not tested the Co (+III) species due to the difficulty of loading of Co (+III) precursor on the MFI zeolite. This can be a subject of the next study.

The TEM and EXAFS thus demonstrated that the Co species was mono‐atomically dispersed on the ion exchange site of MFI, the XANES showed the oxidation state +II, and the ammonia IRMS‐TPD pointed out the Lewis acidity in the region of Co/Al<0.6. Excess Co formed aggregates of CoO. From the reaction tests, the methylation of benzene with methane (1) proceeded on Co/MFI with Co/Al<0.6. The further loading decreased activity for the desired reaction (1) and kept increasing the activity of side reaction (3). These facts derive a conclusion that divalent (oxidation state +II) cobalt species with Lewis acidity mono‐atomically dispersed on the ion exchange site of MFI zeolite was the active site for the methylation of benzene with methane.

It has been found that oxidized states of metals such as Ag^27^ and Cu^38^ loaded on MFI were active for dehydrogenation of methane into intermediate species. We presently carried out the reaction at 773 K in methane, i. e., strongly reductive conditions, toward to achieve practical reaction rate. It is speculated that Co kept its oxidation state even in the present conditions, as shown by XANES, because it was insensitive to the conditions compared to Ag and Cu, and this may be one of the reasons for the high activity of Co/MFI.

## Conclusions

Benzene was methylated with methane in non‐oxidative conditions at 773 K on Co/MFI zeolite as the catalyst. The activity was created by loading of Co on MFI, and the maximum activity was observed with appropriate Co/Al molar ratio 0.6. High concentration of Al, when Co/Al ratio was fixed at 0.6, resulted in the high activity. The isotope and MS experiments evidenced the reaction formula. The ammonia IRMS‐TPD indicated that the loaded Co species were Lewis acidic and mainly held by the ion exchange sites of MFI zeolite. The TEM and EXAFS showed that Co species were mono‐atomically dispersed at Co/Al<0.6. The XANES showed the oxidation state of Co to be +II. The active site for the methylation of benzene with methane is suggested to be the Co (+II) species with Lewis acidity mono‐atomically dispersed on the ion exchange site of MFI zeolite.

## **Experimental Section**

### Catalyst Preparation

Samples of Na‐MFI zeolite with SiO_2_/Al_2_O_3_=24 (supplied by Tosoh), 30, 48 (Mizusawa) and 60 (Zeolyst) were ion‐exchanged into NH_4_‐form, and in addition, a sample of NH_4_‐form MFI with SiO_2_/AlO_2_=22 from Tosoh was also used as the support of transition metal catalysts. Various elements were impregnated from aqueous solutions of their nitrates (Co, Fe, Ni, Cu, Ag and In), chlorides of ammine complexes (Pt, Pd and Rh) or (NH_4_)_6_Mo_7_O_24_ on NH_4_‐MFI. Most of the solvent was removed by drying at 343 K with stirring at 400 rpm. The yielded solid was dried again at 383 K overnight in an oven and then stored without further calcination at higher temperatures. Cobalt was also impregnated on NH_4_‐*BEA (ion exchanged from Na‐*BEA, Clariant), NH_4_‐MOR (as supplied from Tosoh) and NH_4_‐FAU (ion exchanged from Na‐FAU, JGC Catalysts and Chemicals) with SiO_2_/Al_2_O_3_=25, 19, and 4.8, respectively. In the case of Co/MFI, an ion exchange method was also examined for the catalyst preparation as follows: NH_4_‐MFI with SiO_2_/Al_2_O_3_=22 was put into an aqueous Co(NO_3_)_2_ solution with desired Co content, and the solution was stirred and heated at 343 K for 4 h. The solid was then filtrated, washed 3 times and dried at 383 K. The Co and Al contents on the ion‐exchanged samples were measured by means of inductively coupled plasma emission spectroscopy (ICP‐AES, Rigaku ICP CIROS). As a comparison, Co/SiO_2_ was prepared by the impregnation of Co(NO_3_)_2_ on a silica gel at [Co]=0.78 mol kg^−1^, which is equivalent to the Co content in Co/MFI prepared by the impregnation at Co/Al=0.6 on the MFI with SiO_2_/Al_2_O_3_=22. The thus prepared samples are listed in Table S1 with their abbreviations.

### Reaction Tests

Catalytic tests were performed in a fixed‐bed flow reactor. Methane (99.9 % from Iwatani) and benzene (special grade, Wako) were used, and in some cases, methane enriched with ^13^C (^13^C 99.9 % from Hinomaru Industry, Tottori) was used for the confirmation of reaction path. In standard conditions, powder sample (0.300 g) was placed in a Pyrex tube (i.d.: 10 mm) and pretreated in a flow of nitrogen (1.23 mmol min^−1^) in the atmospheric pressure at 823 K for 1 h. Then, a mixture of methane and benzene (98.6 and 2.7 kPa, 1.2 and 0.033 mmol min^−1^, respectively, corresponding to *W*_cat_/*F*_benzene_=147 g_cat_ h mol_benzene_^−1^) was fed to the catalyst bed at 773 K. The outlet materials were trapped by hexane at 273 K with 1,4‐diisopropylbenzene as an inner standard material and analyzed with flame ionization detector‐gas chromatograph (FID‐GC, Shimadzu GC‐2010) or were analyzed by using a mass‐spectrometer (MS, Pfeiffer Vacuum QMS200) directly connected to the outlet of a reactor. The MS measurements were carried out by means of inner standard method using helium as the standard. The molecular weight of the product of reaction using ^13^C‐enriched methane was analyzed with a GC‐MS (JMS‐T100GCV, JEOL). The ^13^C NMR were recorded on JEOL JNM ECP500 at 11.7 T. Chemical shifts are expressed in ppm downfield from Si(CH_3_)_4_.

### Analysis of Physicochemical Properties

The acidic property was analyzed by a method of ammonia IRMS‐TPD in conditions described elsewhere.^42^ Morphology of the catalyst was analyzed with a TEM (HITACHI H800 B) in accelerating voltage 200 kV. Oxidation state and microstructure of the Co species were analyzed by X‐ray absorption spectroscopy (XAS or XAFS, i. e., XANES and EXAFS) at BL01B1 in Japan Synchrotron Radiation Research Institute (JASRI, SPring‐8) (Proposal No. 2018A1075). After pretreatment of Co/MFI in nitrogen flow of 74 mmol h^−1^ at 101 kPa and 823 K for 1 h, it was mixed with boron nitride (BN), stirred by an agate mortal for 30 min and compressed into a wafer form with 10 mm diameter. A Co‐foil and bulk Co oxides (CoO and Co_2_O_3_) were also measured as the references. The Co K‐edge absorption spectra were collected in the quick mode using a Si (111) monochromator. The beam size at the sample position was 5 mm (horizontal)×1 mm (vertical).

## Conflict of interest

The authors declare no conflict of interest.

## Supporting information

As a service to our authors and readers, this journal provides supporting information supplied by the authors. Such materials are peer reviewed and may be re‐organized for online delivery, but are not copy‐edited or typeset. Technical support issues arising from supporting information (other than missing files) should be addressed to the authors.

Supplementary
