# Supplementary material for: Direct Methylation of Benzene with Methane Catalyzed by Co/MFI Zeolite
Source: ChemCatChem. 2018 Jul 11;10(17):3806–12. doi: 10.1002/cctc.201800724 (PMC6282706; doi:10.1002/cctc.201800724)
Supplement: Supplementary file 1 — Supplementary [file CCTC-10-3806-s001.pdf]

Supporting Information

© Copyright Wiley-VCH Verlag GmbH & Co. KGaA, 69451 Weinheim, 2018

Direct Methylation of Benzene with Methane Catalyzed by Co/MFI Zeolite

Koshiro Nakamura,* Akihito Okuda, Kiyotaka Ohta, Hitoshi Matsubara, Kazu Okumura, Kana Yamamoto, Ryosuke Itagaki, Satoshi Suganuma, Etsushi Tsuji, and Naonobu Katada

Table S1. Catalysts employed in this study

Abbreviation	Transition metal source	Preparation method	Structure of support	[Al] / mol kg^{-1}	[Metal] / mol kg^{-1}	SiO_2/Al_2O_3 in support	Metal / Al molar ratio
H/MFI	-	-	MFI	1.3	0	22	0
IMP-Co-*x*	$Co(NO_3)_2$	Impregnation	MFI	1.3	1.3 × *x**	22	*x**
IE-Co-*x*	$Co(NO_3)_2$	Ion exchange	MFI	1.3	1.3 × *x***	22	*x***
Co/MFI (24)	$Co(NO_3)_2$	Impregnation	MFI	1.3	0.78*	24	0.60*
Co/MFI (30)	$Co(NO_3)_2$	Impregnation	MFI	1.0	0.61*	30	0.60*
Co/MFI (48)	$Co(NO_3)_2$	Impregnation	MFI	0.7	0.39*	48	0.60*
Co/MFI (60)	$Co(NO_3)_2$	Impregnation	MFI	0.5	0.32*	60	0.60*
Co/BEA	$Co(NO_3)_2$	Impregnation	BEA	1.2	0.72*	25	0.60*
Co/MOR	$Co(NO_3)_2$	Impregnation	MOR	1.6	0.95*	19	0.60*
Co/FAU	$Co(NO_3)_2$	Impregnation	FAU	4.5	2.71*	4.8	0.60*
Co/SiO_2	$Co(NO_3)_2$	Impregnation	Amorphous (silica gel)	0	0.78*	∞	∞
Fe-0.6	$Fe(NO_3)_3$	Impregnation	MFI	1.3	0.81*	22	0.60*
Ni-0.6	$Ni(NO_3)_2$	Impregnation	MFI	1.3	0.81*	22	0.60*
Cu-0.6	$Cu(NO_3)_2$	Impregnation	MFI	1.3	0.81*	22	0.60*
Zn-0.6	$Zn(NO_3)_2$	Impregnation	MFI	1.3	0.81*	22	0.60*
Mo-1.2	$(NH_4)_6Mo_7O_{24}$	Impregnation	MFI	1.3	1.62*	22	1.20*
Rh-0.6	$RhCl_3$	Impregnation	MFI	1.3	0.81*	22	0.60*
Pd-0.6	$PdCl_2$	Impregnation	MFI	1.3	0.81*	22	0.60*
Ag-0.6	$AgNO_3$	Impregnation	MFI	1.3	0.81*	22	0.60*
In-0.4	$In(NO_3)_3$	Impregnation	MFI	1.3	0.54*	22	0.40*
Pt-0.6	$H_2[PtCl_6]$	Impregnation	MFI	1.3	0.81*	22	0.60*
H/MFI	-	-	MFI	1.3	0	22	0

*: Based on the amounts of Co in the impregnated solution and Al in the used zeolite.

**: Measured by inductively coupled plasma emission spectroscopy (ICP-AES).

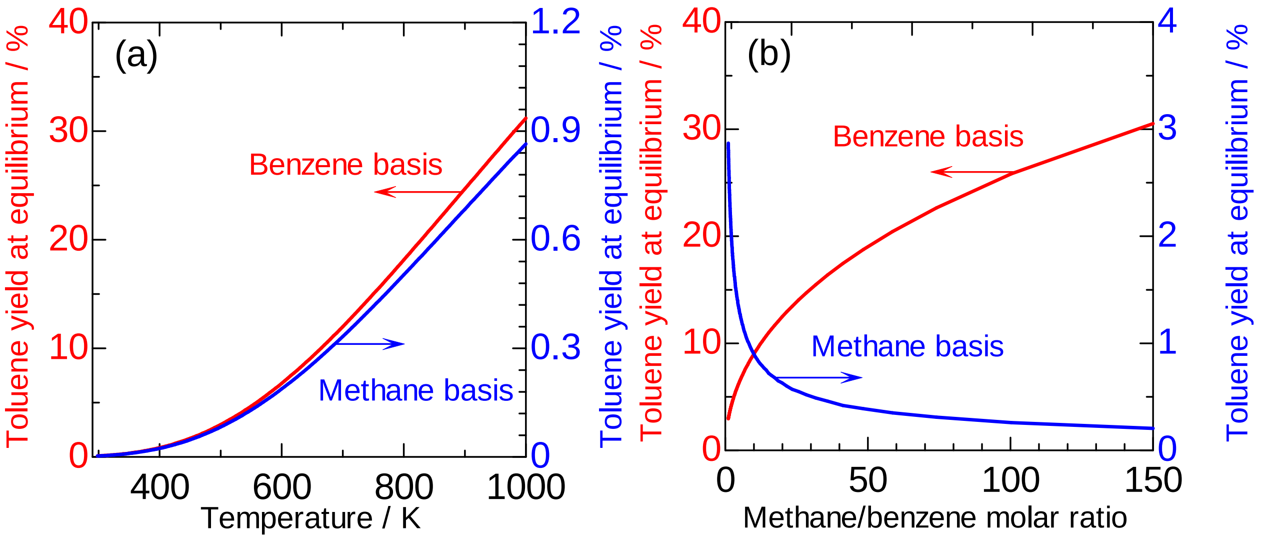

Figure S1.

Figure S1. Plots of toluene yield at equilibrium calculated from thermodynamic parameters and normalized by feeds of benzene and methane (a) against temperature with fixing methane/benzene molar ratio at 36.5 and (b) against methane/benzene molar ratio with fixing temperature at 773 K. Because this reaction does not change the number of gaseous molecules, the total pressure does not affect the equilibrium.

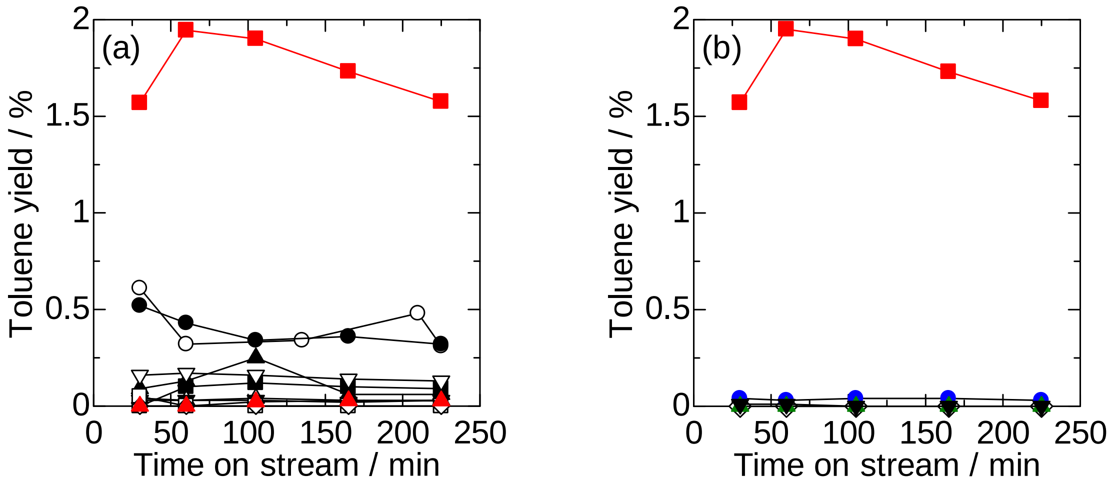

Figure S2.

Figure S2. Time course of toluene yield at 773 K, P_{CH4} = 98.6 kPa, P_{C6H6} = 2.7 kPa and W_{cat} / $F_{benzene}$ = 147 g_{cat} h $mol_{benzene}^{-1}$, over (a) Co (■, IMP-Co-0.6), H (▲, H/MFI), Ni (●, Ni-0.6) , In (○, In-0.4), Mo (▲, Mo-1.2), Cu (■, Cu-0.6), Ag (△, Ag-1.2), Fe (▽, Fe-0.6), Pt (▼, Pt-0.6), Pd (□, Pd-0.6) and Rh (◇, Rh-0.6) impregnated on MFI (SiO_2/Al_2O_3 = 22), where the digits in parentheses show metal / Al and (b) Co impregnated on MFI (■, IMP-Co-0.6), BEA (●, Co/BEA), MOR (▼, Co/MOR), FAU (▲, Co/FAU) and silica gel (◇, Co/SiO_2) with keeping Co/Al at 0.6 on zeolites (■, ●, ▼ and ▲) while [Co] was 0.78 mol kg^{-1} on silica gel (◇).

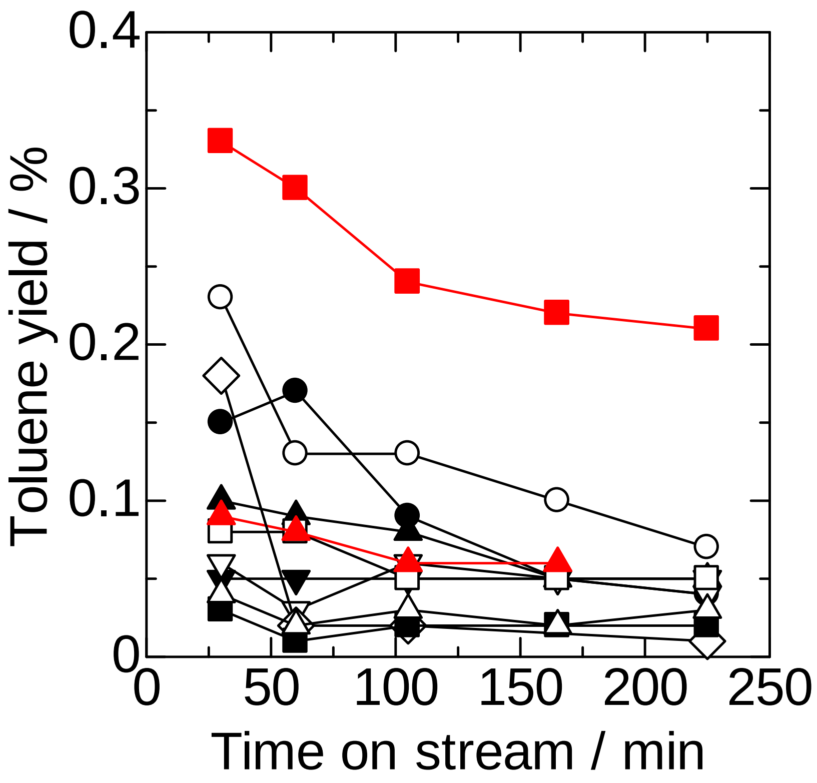

Figure S3.

Figure S3. Time course of toluene yield under high pressure (P_{CH4} = 1200 kPa and P_{C6H6} = 290 kPa) at 773 K and W_{cat} / $F_{benzene}$ = 5.7 g_{cat} h $mol_{benzene}^{-1}$, over (a) Co (■, IMP-Co-0.6), H (▲, H/MFI), Ni (●, Ni-0.6) , In (○, In-0.4), Mo (▲, Mo-1.2), Cu (■, Cu-0.6), Ag (△, Ag-1.2), Fe (▽, Fe-0.6), Pt (▼, Pt-0.6), Pd (□, Pd-0.6) and Rh (◇, Rh-0.6) impregnated on MFI (SiO_2/Al_2O_3 = 22), where the digits in parentheses show metal / Al.

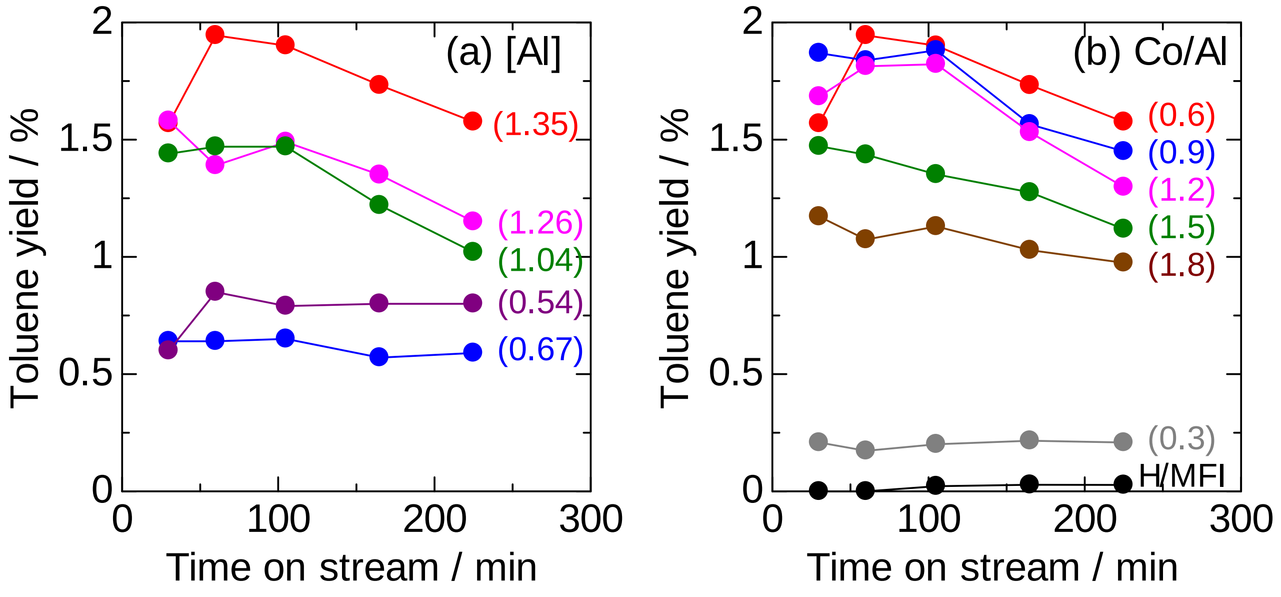

Figure S4.

Figure S4. Time course of toluene yield over Co impregnated on (a) MFI (SiO_2/Al_2O_3 = 22) with various Co contents (digits in parentheses: Co/Al molar ratio) and (b) MFI with various Al contents with Co/Al = 0.6 (digits in parentheses : [Al] / mol kg^{-1}) at 773 K, P_{CH4} = 98.6 kPa, P_{C6H6} = 2.7 kPa and W_{cat} / $F_{benzene}$ = 147 g_{cat} h $mol_{benzene}^{-1}$.

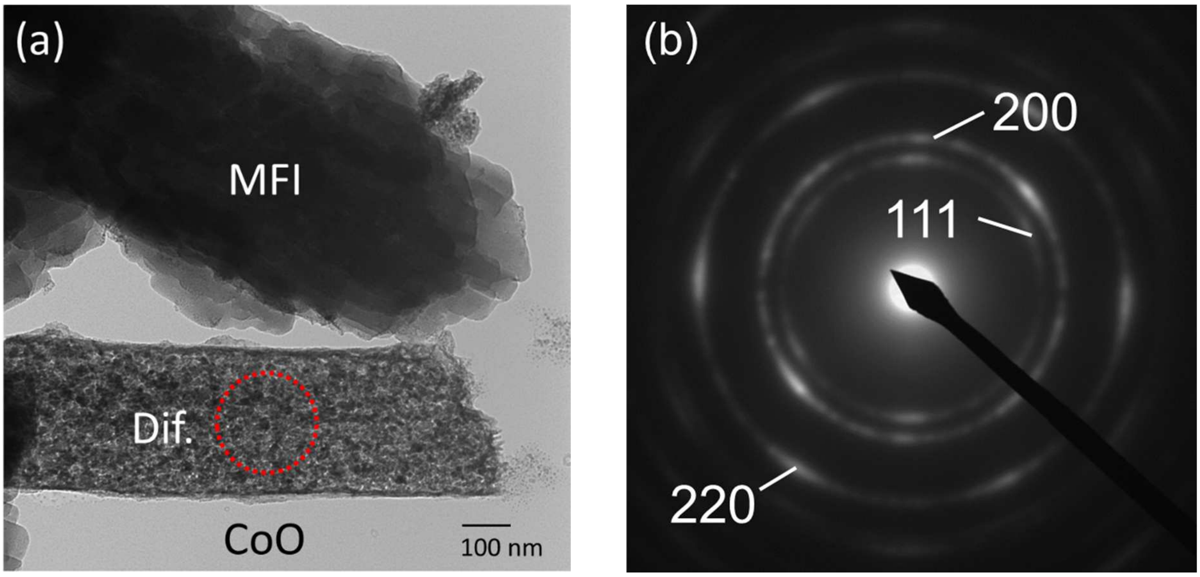

Figure S5.

Figure S5.　(a) TEM image of IMP-Co-1.8, and (b) electron diffraction pattern at point shown by red circle in Figure S4 (a).
